# Cerebrating and engagement, paths to reduce fresh produce waste within homes

**DOI:** 10.1038/s41598-024-58250-0

**Published:** 2024-04-06

**Authors:** Cathrine V. Jansson-Boyd, Cari-lène Mul, Daniela Raeva-Beri

**Affiliations:** 1 School of Psychology and Sport Science, Anglia Ruskin University, Cambridge, UK; 2 Lord Ashcroft Business School, Anglia Ruskin University, Cambridge, UK

**Keywords:** Psychology, Environmental sciences

## Abstract

A real-world study was conducted with the aim to reduce people’s fresh fruit and vegetables waste within their homes. For 6 weeks participants measured their fresh produce waste. Half the participants were impelled to complete food waste logs whilst the other half was a control group. This was followed by a 6-month monitoring period to establish if changes would last. Fresh produce waste decreased with over a quarter of what the participants had wasted at the beginning of the 6 weeks, for all groups. Additionally, an attitude questionnaire distributed at the onset and at the end of the study showed a shift in pro-reduction of food waste. As this indicated that thinking about food waste prompts engagement, we tested this idea using a different sample group. A questionnaire measuring attitudes and cognition confirmed the importance of thinking and provided further insight into the findings from the first study.

## Introduction

This research aimed to create a functionable framework that can help to establish positive attitudes to the reduction of foodwaste as well as actual reduction of foodwaste. Foodwaste is a serious issue as it has wide reached implications for food security, environmental sustainability, and climate change^1^. UNEP’s Food Waste Index^2^ suggests that in 2019, around 931 million tonnes of foodwaste were generated, of which 61% came from households. For every 1 kg of edible food wasted, approximately 2.5 kg CO_2_ is emitted^3^ which in total accounts for 8% of all greenhouse gas emissions^2^. As a comparison, it is approximately four times more than the greenhouse gas emissions produced by the aviation industry^4^.

Reducing foodwaste can help develop a sustainable food system and subsequently combat global hunger and reduce associated deaths^5^. Unfortunately, governments have failed to communicate effectively why people should reduce their waste^6,7^. Hence, foodwaste within homes is rife^8^ and can account for as much as 1 kg of food a week^9^ even though many are uneasy about wasting food^9–13^.

Approximately 4.9 million tonnes of the food thrown away, can be eaten^14^. Most commonly people waste fresh produce (i.e., fruit and vegetables)^15,16^, which is why it is the focal point of this research. In the UK, fresh produce makes up 46% of wasted edible food by weight and this is around 64 kg per household per year^17^. This translates into 35% of all the greenhouse gases emissions that results from food waste produced by UK households^17^. Based on an estimate of 28.2 million households^18^, it means that wasted fresh produce emits over 4500-ton CO_2,_ which is more than a large carbon dioxide removal plant can capture in a year^19^.

Whilst suggestions on how to reduce food waste can be found, it often lacks strong empirical support^20^ and ecological validity. To overcome the latter, this study was conducted within the participants homes. A recent review of food waste reduction studies^21^ reported that information campaigns was the predominant tool used to encourage food waste reduction. In the first study, some information was given as part of the intervention, but it stands out by aiming to put food waste at the forefront of the mind of the participant when engaging in food related behaviour (e.g., shopping, storing, cooking) by providing daily reminders and aids, and by the duration of the study—long enough to potentially establish new habits.

### Difficulty measuring foodwaste

People are often unaware of how much food they throw away daily^22^ and even when they are aware, they usually don’t know how much or frequently^23^. To raise awareness it is essential to give them methods of reporting that helps them to assess their waste more accurately. As there is no one recognised method that is effective to measure food waste within the home^24^ this project used two different methods, *food diaries*^2–6,8–16,20,22–27^ and reporting of *food waste using a log*^28^. Both measures have previously been deemed suitable to measure waste. Including two different methods, could ensure that the reporting of foodwaste was not hampered by the method used. It also allowed us to observe whether one method worked better or if they were equally effective. Two groups (one intervention group and one control group) used food diaries, and similarly two groups (intervention and control) used food waste logs.

### Limited time and thinking

Time limitation is one reason why people are unaware of how much food they throw away^22^. People frequently lack time when preparing for meals and thus cooking related decisions are often made with little or no thinking^29,30^. Consequently, people tend to make fast, simple, and often repeat decisions. Therefore, food ingredients that require more preparation or ‘creative thinking’ in terms of use, are left on the shelf because of limited cognitive input.

Time limitation also influences how food is stored and hence people are unlikely to reorganise their cupboards with each purchase to ensure systematic stacking of newer and older foods. Systematically storing and categorising food products can lower food waste^8^. However, doing so can be a struggle for people, and thus they require assistance with organisation so that items that need to be eaten can be easily located^8^. This all points towards that it is important to ensure that food waste is put at the forefront of people’s mind, seeming that lack of conscious input makes waste more likely. Consequently, making people consciously aware was a key premise for this research and the interventions were designed to achieve this. Firstly, the intervention groups received daily text reminders to complete their waste log/diary. Secondly, we aimed to simplify the process of identifying fresh produce that needed eating and thus reduce the cognitive effort required during cooking times. We gave participants a chart that logged the produce they purchased and any best-by dates. This chart reminded people of what needed to be used next easing the burden of digging through the fridge for expiration dates on every cooking occasion. Moreover, using the chart created ‘visibility’ as they were asked to put it in a clearly visible place in their kitchen, such as on the fridge. Thus, it would function as an aid to remind people to avoid food waste and making it an integrated household practice and putting it at the forefront of their minds.

### Positive attitudes towards reduction of food waste

Ensuring that something is put to the forefront of people’s minds can help with the strengthening of pro-environmental attitudes. The more extensively someone thinks about a topic, the more likely they are to generate stronger attitudes towards it^31,32^. If a person fails to be conscious of the reasons as to why they waste food it is harder for them to rectify their actions^33^. It has been reported that some important barriers stopping people engaging with reduction of food waste are to do with existing food management behaviours, such as shopping, storing, cooking and eating^34,35^ suggesting that, in order to reduce food waste, unconscious habits might need to be replaced by new habits, Stancu et al.^36^. The interventions were designed both to address food-management behaviour as well as new habit forming^36^.

Theoretically, the Theory of Planned Behaviour (TPB) might explain such changes in behaviour. Therefore, a measure of the TPB was included^37,38^. The theory proposes that the best predictor of a person’s behaviour is their intention to perform that behaviour. The intentions in turn are affected by attitudes (towards reduction of foodwaste), subjective norms (perceived expectations of others and how much the individual values those expectations) and perceived behavioural control (how able an individual feels to perform a specific behaviour).

To find out participants attitudes towards food waste at the onset of the study as well as if they changed after taking part, a questionnaire consisting of five different constructs (ethics, environmental issues, purchase behaviours, food preparation and whether people are affected by food expiration dates) was distributed^39^.

Finally, a relationship between cognition and TPB was explored in an additional survey to the food waste study as results suggested that thinking about wasting food could influence people’s intention to reduce food waste as well as their actual behaviours. The same constructs that were used for the questionnaire in the main study to measure TPB as well as an additional factor measuring cognition, was included. Moreover, 3 factors, self-identity, moral norm, and anticipated regret, were included as they are known to influence intent^40^.

## Results

All assumptions of the statistical tests reported were met. Attitude measures collected at the onset showed both aligned and distanced attitudes towards the topic of this study. Many were concerned about foodwaste during food preparation (61%) and were not overly influenced by expiration dates (61%). Many participants were neutral in their environmental (55%) and ethical (49%) concerns and were not bothered about their purchasing behaviours (40%).

The overall attitude stance at the beginning was compared to attitudes held at the end of the study (mean values for each attitude can be seen in Table 1). All attitudes had increased in a pro-food waste reduction direction. A significant difference was found between the three attitude stances on each of the attitude measures, apart from one (not influenced by expiration date). Also, changes in purchase behaviours were not statistically significant.

**Table 1:** Attitude means.

Attitude measures	Overall		Stance against		Neutral		Care about	
	Before	After	Before	After	Before	After	Before	After
Environment	11.46 (2.26)	11.87 (2.42)*	8.92	8.89	12.17	12.42	14.40	15.41
Ethics	12.01 (2.0)	12.39 (2.23)*	9.65	11.11	12.15	12.37	12.01	14.14
Not influenced by expiration date	14.59 (1.98)	15.27 (1.99)*	9.90	14.30	12.60	13.99	14.59	16.00
Preparation	13.19 (2.47)	13.55 (1.90)*	8.63	11.60	12.41	13.88	15.09	15.00
Purchase behaviours	13.52 (3.09)	13.55 (2.39)	9.16	11.07	12.70	13.40	13.52	14.80

*Indicates that the mean difference was significant at < 0.05. Numbers in brackets are standard deviations and the shaded areas show the means for the different attitude stances.

Additionally, we tested if the change in attitudes depended on whether they were strong caring, neutral or somewhat against food waste reduction at the onset. Results showed that there were distinctly different attitude types among the participants in that there was a difference in ratings between them. Results showed that nearly all attitudes had become more positive after participation, and this was true for all attitude types. Mean values can be seen in the shaded section of Table 1.

### Factors affecting intent

A regression analysis was used to test the variables measured for the TPB (only significant variables are reported here). Looking at *intent*, for the data collected at the beginning, a significant model emerged F(7, 127) = 6.07, *p* < 0.001, R^2^ = 0.230, and adjusted R^2^ = 0.193. The variables that significantly influenced the model was ‘ethical attitudes’ and ‘perceived behavioural control’ (*p* = 0.011).

**Figure 1:**
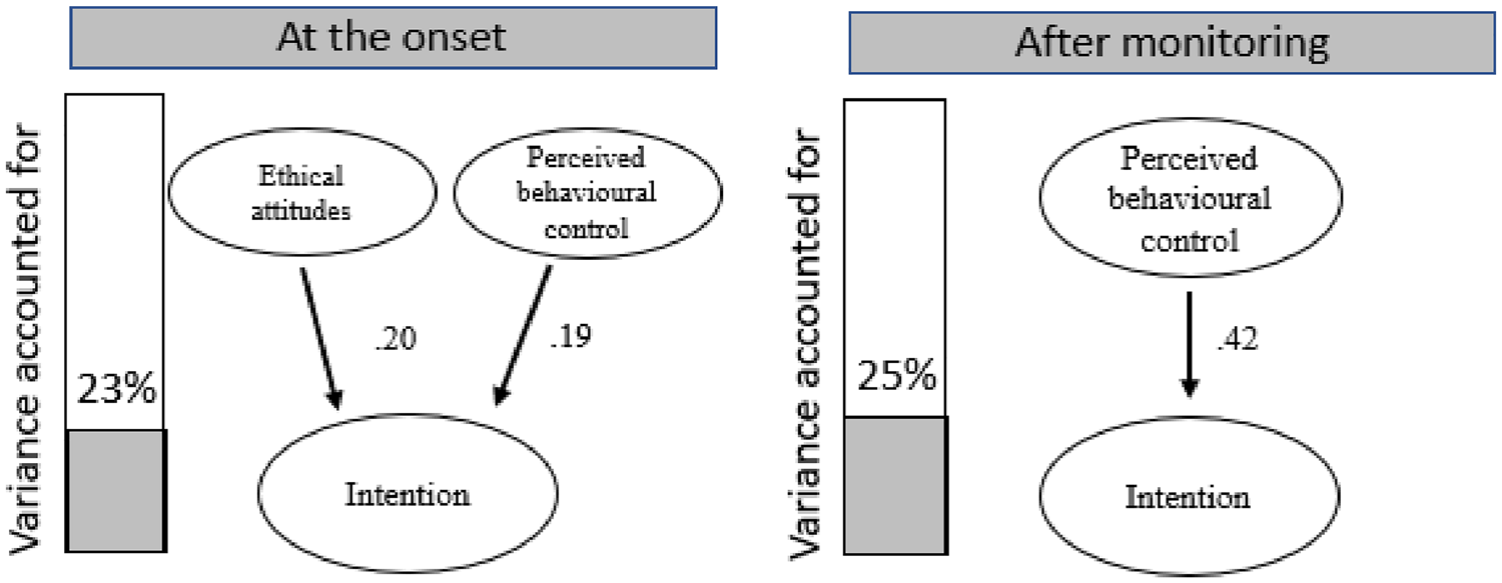
The figure shows the path analysis for intent at the onset of the study (left) and after the monitoring had been completed (right). The bar graph on the left shows the overall variance accounted for in the model. The beta values for the significantly contributing variables are next to the arrows demonstrating how they feed into *intent* to reduce food waste.

A significant model was also found for *intent* after the monitoring period F(7, 127) = 5.77, *p* < 0.001, R^2^ = 0.252, and adjusted R^2^ = 0.208. The only variable that was found to significantly influence the model was perceived behavioural control (*p* < 0.001), see Fig. 1.

Perceived behavioural control doubled in strength after the monitoring period as it accounts for 42% compared to only 19% at the beginning (see Fig. 1). Thus, taking part in the study made participants think they are in control of their foodwaste, and it therefore increases *their intentions* to reduce food waste. Possibly, the fact that they know they can reduce food waste is empowering, as taking part in the study has taught them that they are in control of their foodwaste. Whilst previously they may have thought they could do it (the influence of PBC at the onset) and that they should (the influence of Ethical attitudes at the onset).

### Fresh produce waste measures

**Figure 2:**
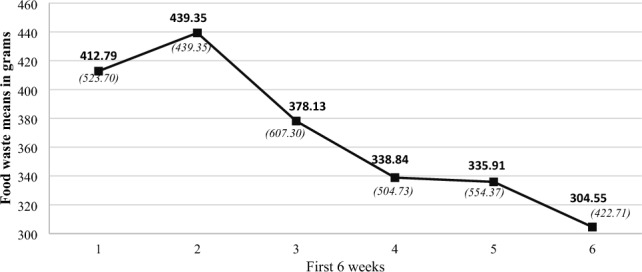
The figure shows the foodwaste for all the participants during the first 6 weeks. The mean values can be seen in bold and *SD* in brackets below. It is noticeable that people cut their waste during the first week and that it increased a bit during the second week. Thereafter there is a continuous decrease in waste amongst *all* the participants until week six.

A repeated measures ANOVA found a significant difference in foodwaste for ***all*** the participants during the first 6 weeks, *F*(1.76, 61.46) = 9.13, *p* = 0.003, η^2^ = 0.02. Means and standard deviations (SD) can be seen in Fig. 2.

Post hoc paired-sample T-tests revealed significant differences between week 1 and week 4; *t* (153) = 2.13, *p* = 0.034, d = 0.17 as well as between week 1 and week 5; *t* (153) = 2.11, *p* = 0.036, d = 0.17. There was also a significant difference between week 1 and 6; t (153) = 3.35, p = 0.001, d = 0.27. Additionally, significant differences were found between week 2 and week 4; *t* (153) = 2.50, *p* = 0.013, d = 0.20 as well as between week 2 and 5;* t* (153) = 2.79, *p* = 0.034, d = 0.23, and week 2 and week 6; *t* (153) = 3.49, *p* < 0.001, d = 0.28. This shows a difference in foodwaste between the beginning and the end of the first 6 weeks. *On the whole participants wasted approximately 108 g per week less fresh produce after the 6 weeks*. This is more than a quarter of what they wasted at the beginning of the 6 weeks.

**Figure 3:**
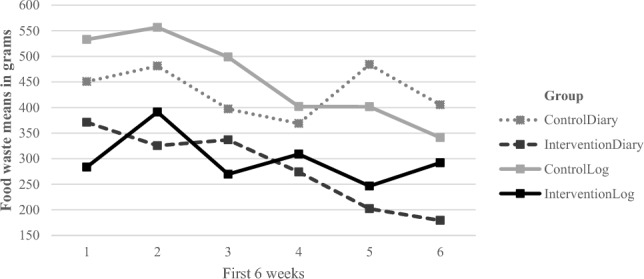
The figure shows the overall waste data of the four different groups during the first 6 weeks. It can be noted that the intervention groups wasted less fresh produce throughout the 6 weeks than those in the control groups. Thus suggesting, that intervention groups benefitted from the interventions.

Independent sample t-tests were used to test if there was a difference between the two different intervention groups and their respective control group, during the first 6 weeks. When comparing the total overall waste, the difference between the diary control group (M = 2586,75, SD = 1957) and the diary intervention group (M = 1682.88, SD = 1612) was significant; *t* (78) = 2.25, *p* = 0.027, d = 0.50, with the diary intervention group wasting significantly less than the diary control group over the course of 6 weeks (Fig. 3).

#### Differences between fruit and vegetable waste

A paired samples *t*-test showed that participants wasted more vegetables (*M* = 1328.7, *SD* = 1558.0) than fruit (*M* = 879.4, *SD* = 1297.6) over the course of 6 weeks, *t* (153) = 5.31, *p* < 0.001, d = 0.42.

Repeated measure Anova’s were conducted separately for wasted fruit and vegetables over the first 6 weeks. It showed that while both types of produce had a reduction in waste, only for fruit was this reduction significant, *F* (4.5, 669.0) = 3.7, *p* = 0.003, d = 0.24. In the first week the mean was 172.9 g and in week six it was 118.0 g (see all means in the supplementary materials, Table 1a and b).

### Monitoring period

**Figure 4:**
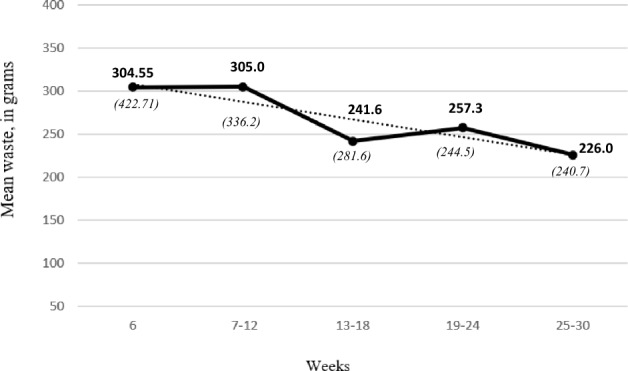
The mean values can be seen in bold and *SD* in brackets below. Week six is the last week of the intervention period. It can be noted from the dotted trend line that the foodwaste during the monitoring period, for all participants, is in line with the reduction implemented during the first 6 weeks. It is also evident that participants continue to reduce their waste.

An average weekly foodwaste was calculated over weeks 7–12, weeks 13–18, weeks 19–24, and weeks 25–30. These were then compared with the level of waste participants had reached in week 6 (baseline). T*-*tests revealed that participants did not significantly increase or decrease their waste in the monitoring period compared to the level they had reached in week 6. Thus, participants successfully maintained their reduced levels of food waste longer term (see Fig. 4).

### Follow on study—results

The attitudes measured in the main study were used to categorise this new set of participants (based on the median) into three groups; those who cared strongly about foodwaste, those who were neutral and those who were somewhat against food waste reduction. Results showed that 54% of participants cared about food preparation and 55% were not influenced by expiration dates. These numbers were also high in the main study (61% for both attitudes). However, there were also some noteworthy differences such as that more negative attitudes towards environmental and ethical reasons to reduce food waste (42% and 89% respectively) was higher than in the main study (25% and 30% respectively). All group comparisons can be seen in the supplementary materials, Table 2.

**Figure 5:**
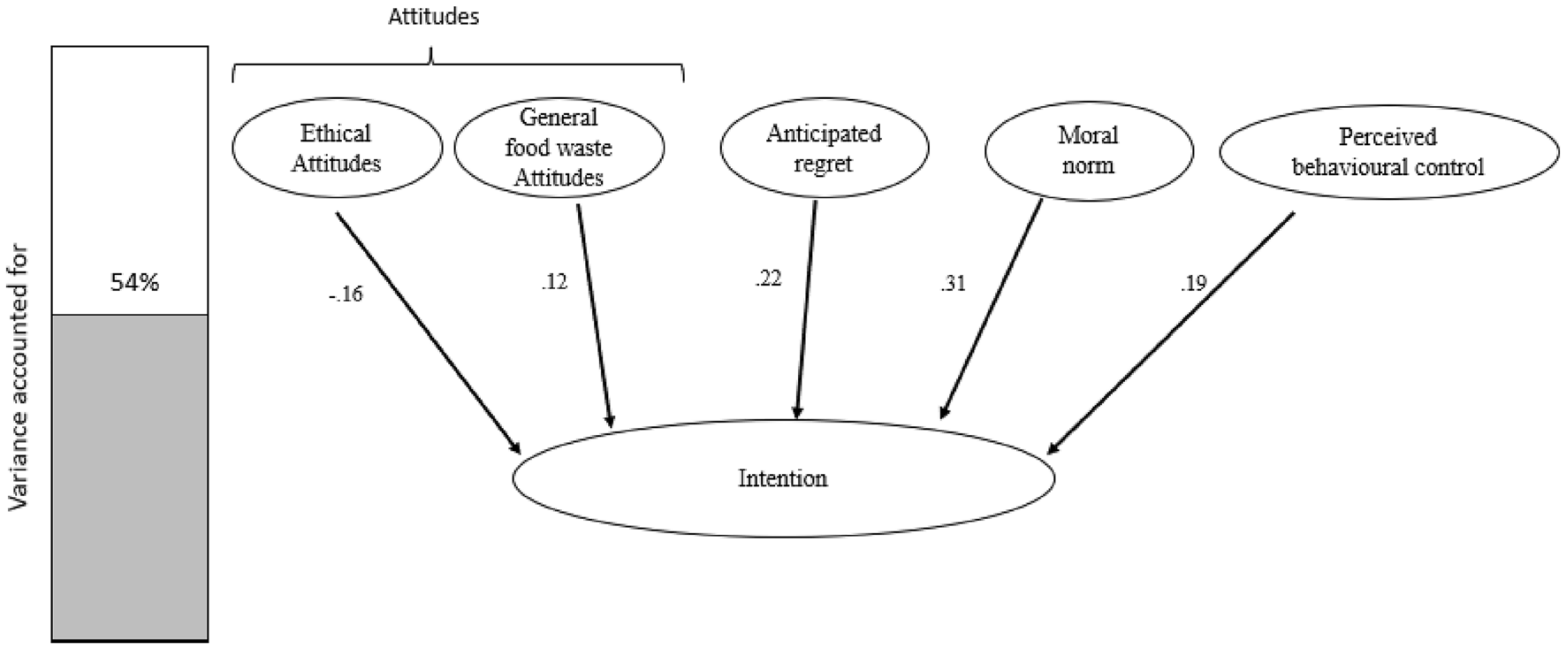
Factors that affect intention to reduce food waste. The bar graph on the left shows the overall variance, the model accounts for 54%. Five factors affect intention to reduce food waste. Two of those are attitude factors (ethics, and general food waste). The other three are anticipated regret, moral norm, and perceived behavioural control. Ethical attitudes are negatively associated with intention whilst all other measures are positive. It can be noted that the strongest measure influencing attention is moral norm.

Regressions were used to test the factors against intention and behaviour. A significant model emerged for intentions F(8, 148) = 20.38, *p* < 0.001, R^2^ = 0.538, adjusted R^2^ = 0.512. Five variables significantly contributed to the overall model (see Fig. 5); ethical attitudes (*p* = 0.02), general attitudes (*p* = 0.05), anticipated regret (*p* = 0.009), moral norm (*p* < 0.001) and PBC (*p* = 0.003). This supports the findings from the main study as it shows that ethically based attitudes have a role to play in people’s intent to reduce food waste. However, it differs from the main food-waste study in that here the beta value was negative. Consequently, for every 1-unit that the Ethical Attitudes increase, the intention will decrease by 16%. This may be the result of having a higher number of participants with attitudes towards ethical reasons to reduce foodwaste. This may suggest that ethical reasons to reduce foodwaste should be used with caution in foodwaste campaigns, as it may be counterproductive for those who are less convinced by ethical reasons.

Consistent with the main study, cognition and subjective norms did not affect the participants intention to reduce self-reported food waste behaviours. It shows that other people’s norms do not significantly influence others in their intention to waste food.

**Figure 6:**
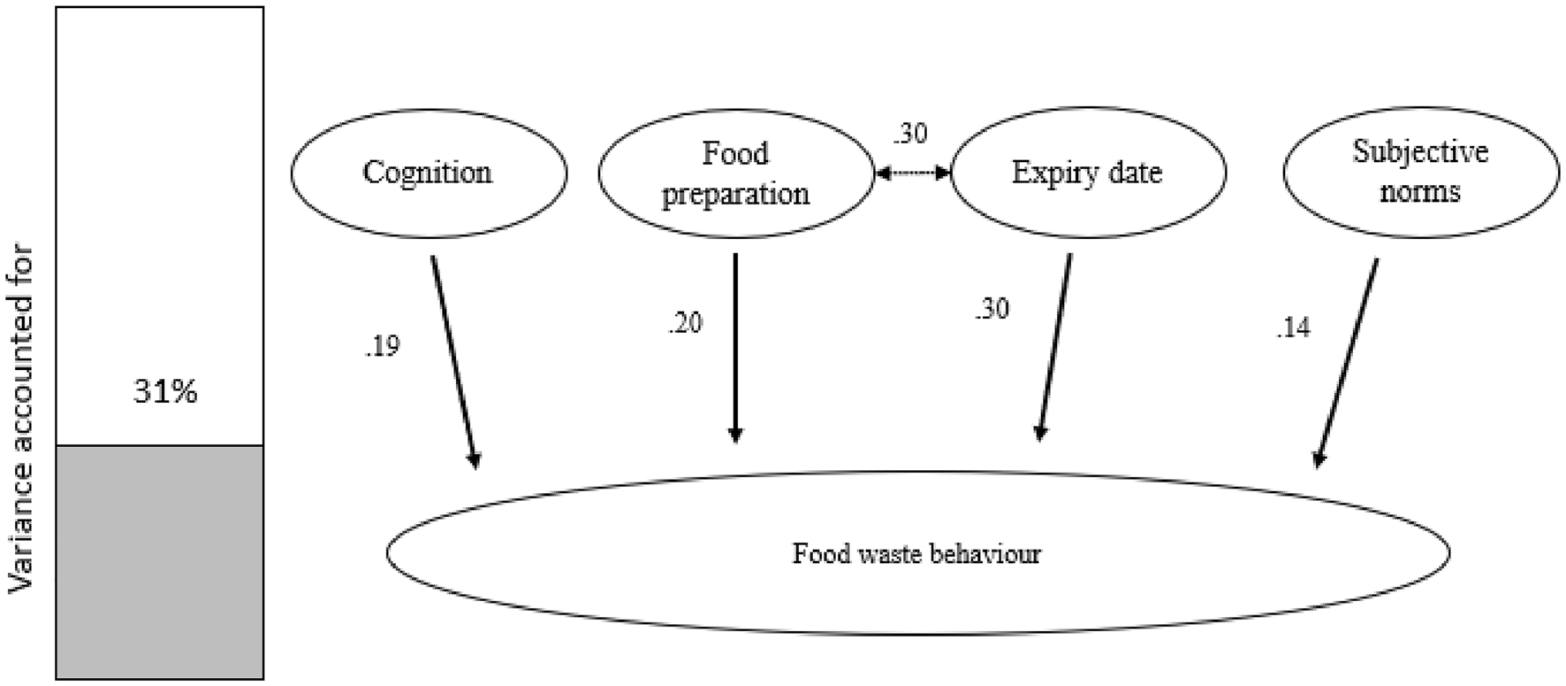
Factors that affect food waste behaviour. The bar graph on the left shows the overall variance that the model accounts for (31%). Self-reported food waste behaviour is affected by four factors, cognition (how much time is spent thinking about food and waste behaviours), food preparation, expiry date and subjective norms. There is also a significant correlation between food preparation and expiry date, indicating that expiration dates to some extent influence diligence to avoid food waste. The expiry date is also the strongest influencing factor on food waste behaviour.

Intention to reduce food waste was not linked to self-reported waste behaviour (also consistent with the foodwaste study). This suggests that intention is comparable to an attitude that does not influence behaviour. However, 4 factors did affect self-reported food waste behaviours (see Fig. 6); cognition (*p* = 0.018), food preparations (*p* = 0.021), expiry date (*p* < 0.001) and subjective norms (*p* = 0.05), F(7, 152) = 8.18, *p* < 0.001, R^2^ = 0.312, and adjusted R^2^ = 0.301.

## Discussion

This project has provided insight into how fresh produce waste within UK homes can be reduced. For 6 weeks participants measured their fresh produce waste. This was followed by a 6-month monitoring period to determine if reductions made during the initial 6 weeks would last. The initial 6 weeks generated an overall decrease, across all groups, over a quarter of what they had wasted at the beginning of the study. This decrease was also found to be maintained during the monitoring period. Additionally, the attitude measure revealed that people became more pro-reduction of waste after having taken part in the study.

The first 6 weeks of the main study also revealed a discrepancy between the reduction of fruit and vegetables. It was found that participants overall wasted more vegetables than they did fruit. However, only the reduction in fruit was found to be significant. There could be several explanations for this, and it is worth exploring in more detail in future studies. A significant fruit waste reduction may be because fruit tends to go off quicker than vegetables, and fruits tend to be eaten as snacks more than vegetables, while vegetables are used more in meals. It may therefore be easier to use up fruit and so reduce fruit waste more than vegetable waste. The fruit waste reduction could also be linked to finances in that people with lower incomes or those undergoing financial difficulties may purchase less to cut down on expenses. If the aim is to reduce waste quickly, it may be worth targeting fruit consumption in the first instance.

The monitoring period showed that participants successfully maintained their reduced levels of food waste over a longer term (see Fig. 4). Thus, suggesting that taking part in a food waste reduction programme can lead to long-term behaviour change. This may be linked to feeling that they are now in control of their food waste. In fact, perceived behaviour control doubled in its influence on intent to reduce food waste. Therefore, giving people the tools to measure their waste in a simple and effective way, empowers them to feel they are in control of their food waste.

As for overall waste, there were no significant differences between waste log and waste diary groups. For future research, for consistency and accuracy, we suggest using only one specific method to log foodwaste as both seems sufficient. However, participants informally reported that the diary method was easier to use. Furthermore, it is worth noting that as with any self-reported data, there is always the possibility of response bias. Hence, future studies should investigate self-reported measures in parallel with actual food waste measures. This would demonstrate accuracy of self-reported measures. Since our studies were conducted during COVID-19, this was not possible to do. There is also the possibility that people will engage in social desirability bias. In this study, it could mean that people report lower amounts of food waste, if they believe it will make them seem more favourable. Thus, this should be considered when looking at the results as it may be that the amount of food waste reduced is higher than recorded.

### Putting it at the forefront of the mind

At the very start, people have a lower amount of waste than in week 2 (see Fig. 2). This may indicate that thinking about and engaging in food reduction processes makes people want to do well, which stops them throwing produce away (which may then go off in week 2). The idea that there is a link between how much time is spent thinking about foodwaste and actual behaviour was confirmed in the follow up study. Cognition was found to be one of four factors contributing significantly to foodwaste behaviours (Fig. 6). Whilst this is a key finding, it should be noted that 69% of the variance is unaccounted for. Hence, it should be explored what additional variables may increase the variance and thus create a better understanding of food waste behaviour.

### Closing remarks

The findings show that all the groups reduced their fresh produce waste, which may be because they all measured their waste. Therefore, it should be explored whether it is the actual action of logging foodwaste that significantly reduces the waste compared to people that don’t. It should also be investigated whether it is the knowledge of being monitored (as people often alter their behaviours when they are monitored^38,40^) or simply taking part in measuring foodwaste that leads to behaviour change. However, as our findings suggests that engaging with the measuring of foodwaste increase people’s feelings of being able to control their foodwaste, we believe that the latter is more influential. When the studies took place may also have influenced the results. COVID-19 and inflation could have contributed to a financially difficult situation. Therefore, people might be motivated to reduce foodwaste to save money. Lastly, there may have been an element of self-selection in our foodwaste study sample—proportionally more participants had positive attitudes towards ethical and environmental reasons to reduce foodwaste than in our follow-up questionnaire study. Also, the high dropout rate may suggest our participants were amongst the most committed to foodwaste reduction.

The work highlights great potential to make use of ‘awareness’ to reduce food waste. Behavioural change is difficult for people as they often engage in routine-based consumer patterns. However, our work shows that this routine can be broken and that it can lead to significant reduction in waste of fresh produce in only 6 weeks. If a 1000 people can be encouraged to reduce their fresh produce waste with 108 g a week, in a year that would be a reduction of over 9.5 tonne CO_2._ This is approximately the same as 1,140,000 smartphone charges, and that is with a relatively small number of people.

## Methods

Our work followed American Psychological Association’s ethical guidelines and as such informed consent was given by all participants. The studies and their procedures were approved by School’s (Psychology and Sport Science) Research Ethics Panel (SREP).

### The foodwaste study

#### Participants

A priori power analysis was calculated using G*Power3^41^ to test the difference between four independent group means using a two-tailed test, a medium effect size (d = 0.50), and an alpha of 0.05. Result showed that a total sample of 256 participants with four equal sized groups of n = 64 was required to achieve a power of 0.80. Thus, the aim was to try and align the participant recruitment figure with the calculation.

Seven hundred and twenty-nine UK based participants were recruited for the first study. A voluntary sampling method was employed. However, as the study was intentionally advertised across the UK, participants recruited were spread across the country. Each participant represented their household. The initial sign-up process included completing an attitude questionnaire towards food waste. Participants were also recruited throughout the project subject to dropout rates.

After the completion of the initial questionnaire, 298 participants confirmed that they would go ahead with the food waste measure. Seventy-nine of those failed to send food-waste logs and a further 64 stopped sending logs during the initial 6 weeks of food waste measure. Twenty-seven participants stopped recording the food waste during the monitoring period. Therefore, there were 127 participants that completed the entire monitoring period across all conditions and a total of 154 participants that completed the entire first 6 weeks and part of the monitoring period.

Participants were divided into one of four groups. There were two groups that both used food diaries and were either in an intervention or control group. The other two groups used the food waste logs and were also divided into an intervention or control group. To ensure that food waste was comparable across the four participant groups, information about the households in which they live was collected, e.g. number of people in the household, age of people in the household, gender, and income (see supplementary material Table 3). The data was used to allocate participants into one of the four study groups. Great care was taken to ensure a similar participant profile across the four groups. Where in the UK participants resided can be seen on Fig. 1, in the supplementary materials.

#### Study structure and questionnaires

There was a main study and an additional study (see method) that were both conducted during the global Covid-19 pandemic. Hence, the design for the study was constructed to ensure that no personal contact was required with participants.

At the onset of the first study participants filled in two questionnaires and then again upon completion. Measuring attitude constructs at the onset and then again after the monitoring period meant that we could measure if any of the attitudes had changed during the participation in the study.

One of the measures broadly reflected attitudes towards food waste^32^ and was subdivided into five specific sub measures, ethics, environmental issues, purchase behaviour, food preparation and whether people are affected by food expiration dates. The ethical measure included questions such as: ‘I feel guilty when I waste food because others don't have enough to eat’ and broadly reflects an ethically conscious mind. Questions related to environmental issues included “Food packaging is a bigger environmental issue than food waste” and reflects whether people consider foodwaste to be an important environmental issue. Purchasing behaviours were tapping into whether participants are conscientious about what they buy, and one question included was: When I see a “Sale” in stores I often buy more than I intended. Similarly, questions related to food preparation also looked at whether people apply diligence to avoid waste. One question in this category was: If something remains after cooking, I freeze it for a later use. Finally, whether people are paying attention to expiry dates was measured by questions such as: I evaluate food to be thrown by its appearance/smell. Hence, tapping into how comfortable people are in making independent decisions regarding the suitability of food use.

The second questionnaire measured the theory of planned behaviour (TPB) which is a theory that links what people believe to behaviour. The theory proposes that there are three aspects that affect a person’s behavioural intentions, attitudes (which in this case was measured as five dimensions, as stated above), subjective norms (an individual's perception of a behaviour, which is influenced by the judgment of others) and perceived behavioural control (which is whether a person perceives a behaviour as being difficult or easy to perform)^39^.

The attitude measures were followed by two different stages. Firstly, by a 6-week period, whereby half the number of participants were subjected to food waste interventions and measured their fresh produce waste. Whilst the other half of the participants only measured their fresh produce waste and therefore acted as a control group. The second stage was a monitoring period whereby all participants measured and recorded their fresh produce waste, but no interventions took place. The waste was reported on a weekly basis. This period was used to find out if the interventions would contribute to a longer-lasting reduction of fresh produce waste. Due to participants dropping out, some at the latter stages of the study, new participants were recruited. This meant that not for all the participants the monitoring periods lasted a full 6 months.

#### Food waste interventions

Participants in the two intervention groups were sent an electronic ‘purchase log’ (see Fig. 2, supplementary material) to fill in for their fresh food upon delivery. Attached to the purchase log was recommendations for when to consume fruit and vegetables safely and whether the items could be frozen, should it not be used by its use by date. When the log was completed, it provided participants with an easy overview that stated on a day-by-day basis what they needed to use to avoid wasting food. All participants were recommended to print the chart off and put it at the front of the fridge, so that it was always visible and thus acted as a constant reminder.

In addition to the purchase log participants received a daily text message, reminding them to check the chart and add any newly bought fresh produce. It also asked them to use any items (or if suitable put them in the freezer) that were due to expire. The text messages were sent by using a scheduling app called Click Send. This was done to ensure that they are all delivered at the same time every day (6 pm).

The interventions were used for the entire 6 weeks of the first stage of the study. As food related routines are something that are created over time, it was anticipated that 6 weeks should be enough to make it part of their daily routines to check food expiration dates and engage in an efficient reduction of food waste practice. It has been found that the creation of routines varies but requires at least 18 days^42^. This being because food-related routines are much influenced by the skills and confidence that consumers have in their abilities to perform activities that help with food waste^43^. Thus, using the chart helps to acquire simple strategies that gives an easy oversight of food use and skills to deal with it.

#### Food waste measures

People are often aware that they waste food, but not how much or frequently^20^ thus it is essential to give them methods of reporting that helps them to assess their waste more accurately. As there is no one recognised method that is effective to measure food waste within the home^22^, this project used two different methods, food diaries and reporting of food waste using a log. Both measures were identified as having the capcity to measure waste correctly compared to some other measures since this study took place during COVID-19 it was essential that the mesures used were both realiable and could be used independently by the participants. However, it is worth noting that beahvioural reactivity in that people waste less during the period of using logs and diaries, as well as misreporting, meaning that people don’t record all the foodwaste generated, ar common problems^44,45^.

Including two different methods, could ensure that the reporting of foodwaste was not disadvantaged by the methodology used. It also meant that we could observe whether one method was superior or whether they produced equal outcomes.

The group using the food diary (see supplementary material, Fig. 3) was asked to log how much food they wasted, in grams. The diary was supplied electronically and dates for recording was put in for ease of use. Participants were asked to record the foodwaste on a daily basis. Keeping a food diary can be a taxing task. Thus, participants were asked to report the waste twice weekly to ensure that they did so accurately whilst not finding it too demanding.

The group reporting food waste through the use of a log (see supplementary materials, Fig. 4), were also asked to bi-weekly indicate the food categories in which waste had occurred. This was done by using a list of categories that was supplied by the researchers. Additionally, participants indicated for each category, the amount of waste in appropriate units. Participants were instructed to report the number of pieces of fruit they wasted (e.g. 2 bananas, half an apple), and with smaller fruits such as berries to report them in bowlfuls. For vegetables participants were asked to report the number of serving spoons wasted. These units were later converted into grams by the researchers using the following guidelines: One piece of fruit (or bowlful) equated to 100 g. One serving spoon of vegetable equated to 50 g. One serving spoon of potato equated to 60 g. This way of measuring food waste was found by the EU initiated Refresh project^28^ to have good validity in that it correlates with other more intensive methods of data collection such as weighing food waste. The main difference between the two methods might be in ease of use. The waste log with reports of items and portions might be easier for the participant than reporting in grams which requires weighing.

Both groups were sent an accompanying leaflet that stated how to fill in the food waste diary. This was to ensure that there was consistency amongst the participants and that it could be referred to throughout the study, if required. Self-report measures based on how much food people think they waste commonly correlate with actual measures of food waste, albeit weakly^46,47^. This shows that people have some insight into what they waste. As we here asked participants to report what they are wasting, rather than estimate, their reports are more likely to be accurate.

### Additional data collection: *link between cognition and food waste*

Based on the findings from the food waste study we wanted to further explore the relationship between cognition and the model of TPB. Thus, it was investigated to what extent *thinking* about wasting food could influence people’s intention to waste food as well as their actual behaviours.

### Participants

A priori power analysis was also calculated for this study, once more using G*Power3 (Faul et al., 2007) a two-tailed test, a medium effect size (d = 0.50), and an alpha of 0.05. Result showed that a total sample of 128 participants was required to achieve a power of 0.80. Here we recruited 154 participants using a voluntary sampling method.

The study was specifically advertised across different UK regions and thus those recruited were from a wide range of locations. There were 101 women and 49 men, aged between 19 and 80 and the mean age was 42. Two people were educated to primary school education, 22 to high school level, 94 to undergraduate university level, and 33 to postgraduate level.

Participants were varied in terms of their income. Twenty were unemployed, 31 people earned below £14, 999, 61 had an income between £15,000 and 34,999, 25 people earned between £35,000 and 54,999. Additionally, 11 people earned between £55,000 and 74,999 and 6 people over £75,000.

Seventy-six percent of all the participants stated that they were the person who did most of the cooking in their household and 78% that they did most of the food shopping. Nineteen percent of the participants lived own their own, 36% lived with one other person, 14% lived with two other people, 20% lived in a household of 4 people, and 8% in a household of 5 people or more. Three percent declined to provide the information.

### What was done

The same constructs that were used for the questionnaires in the main study to measure TPB were also used here. To investigate how thinking more extensively about food waste (cognition) may have a role to play in TPB an additional factor measuring this was included. The cognition factor consisted of 4 questions and were all measured on a five-point Likert scale.

Additionally, 3 factors that have been identified as being predictors of intention^30^ were included, and they were self-identity, moral norm, and anticipated regret. *Self-identity* is about considering if food waste fits with who you think you are. This was measured by the question ‘I am the type of person who would reduce the amount of fruit and vegetables that gets thrown away from my household over the next seven days’. *Moral norm* relates to how a person perceives a particular behaviour to be correct. This was measured by the question ‘I feel a strong obligation to reduce the amount of fruit and vegetables that gets thrown away from my household over the next seven days’. *Anticipated regret* is about your own awareness of whether you may regret wasting food. This was measured by the question ‘I would feel regret if I did not reduce the amount of fruit and vegetables that gets thrown away from my household over the next seven days’.

### Supplementary Information

Supplementary Information.

## Data Availability

The datasets used and analysed during the current studies are available from the corresponding author on reasonable request.

## References

[1] Schanes K, Dobernig K, Gözet B (2018). Food waste matters-A systematic review of household food waste practices and their policy implications. J. Clean. Prod..

[2] UNEP Food Waste Index. Retrieved from: https://www.unep.org/resources/report/unep-food-waste-index-report-2021 (2021)

[3] UN,  (2013). Food Wastage Footprint Impact on Natural Resources.

[4] Szymkowiak A, Borusiak B, Pierański B, Kotyza P, Smutka L (2022). Household food waste: The meaning of product’s attributes and food-related lifestyle. Front. Environ. Sci..

[5] FAO. Retrieved from: https://www.fao.org/3/i3347e/i3347e.pdf (2011)

[6] Hebrok M, Boks C (2017). Household food waste: Drivers and potential intervention points for design–An extensive review. J. Clean. Prod..

[7] Kim J, Rundle-Thiele S, Knox K, Burke K, Bogomolova S (2020). Consumer perspectives on household food waste reduction campaigns. J. Clean. Prod..

[8] Hebrok M, Farr-Wharton G, Foth M, Choi JHJ (2014). Identifying factors that promote consumer behaviours causing expired domestic food waste. J. Consum. Behav..

[9] Kim J, Rundle-Thiele S, Knox K, Burke K, Bogomolova S (2020). Consumer perspectives on household food waste reduction campaigns. J. Clean. Prod..

[10] McCarthy B, Liu HB (2017). Food waste and the ‘green’consumer. Australas. Mark. J..

[11] Stefan V, van Herpen E, Tudoran AA, Lähteenmäki L (2013). Avoiding food waste by Romanian consumers: The importance of planning and shopping routines. Food Qual. Prefer..

[12] WRAP. Retrieved from: https://wrap.org.uk/taking-action/food-drink (2021)

[13] De Laurentiis V, Corrado S, Sala S (2018). Quantifying household waste of fresh fruit and vegetables in the EU. Waste Manag..

[14] Conrad Z (2020). Daily cost of consumer food wasted, inedible, and consumed in the United States, 2001–2016. Nutr. J..

[15] Reynolds C, Goucher L, Quested T, Bromley S, Gillick S, Wells VK, Evans D, Koh L, Kanyama AC, Katzeff C, Svenfelt Å (2019). Consumption-stage food waste reduction interventions–What works and how to design better interventions. Food Policy.

[16] EPA. United States Environmental Protection Agency; https://www.epa.gov/recycle/preventing-wasted-food-home (2022)

[17] WRA. Household Food and Drink Waste in the United Kingdom 2021–22, Retrieved from: https://wrap.org.uk/sites/default/files/2023-11/Household%20Food%20and%20Drink%20Waste%20in%20the%20United%20Kingdom%202021-22.pdf (2021)

[18] Office for National Statistics. Families and households in the UK, from: https://www.ons.gov.uk/peoplepopulationandcommunity/birthsdeathsandmarriages/families/bulletins/familiesandhouseholds/2022#:~:text=Households-,There%20were%20an%20estimated%2028.2%20million%20households%20in%20the%20UK,2012%20(26.6%20million%20households (2022)

[19] Masterson, V. Companies are sucking carbon from the atmosphere using ‘direct air capture’. How does it work? Downloaded from: https://climatechampions.unfccc.int/companies-are-sucking-carbon-from-the-atmosphere-using-direct-air-capture-how-does-it-work/ (2022)

[20] Ganglbauer, E., Fitzpatrick, G., & Molzer, G. Creating visibility: understanding the design space for food waste. In *Proceedings of the 11th International Conference on Mobile and Ubiquitous Multimedia* 1–10 (2012).

[21] Kim J, Rundle-Thiele S, Knox K (2019). Systematic literature review of best practice in food waste reduction programs. J. Soc. Mark..

[22] van Herpen, E., van der Lans, I. A., Nijenhuis, M. A., Holthuysen, N. T. E., & Kremer, S. Best practice measurement of household level food waste: Milestone no. 2. EU. (2016).

[23] Quested TE, Palmer G, Moreno LC, McDermott C, Schumacher K (2020). Comparing diaries and waste compositional analysis for measuring food waste in the home. J. Clean. Prod..

[24] Food waste stories. Retrieved from: https://foodwastestories.com/2020/09/29/how-is-food-waste-measured-and-why-does-it-matter/ (2020)

[25] Djekic I, Miloradovic Z, Djekic S, Tomasevic I (2019). Household food waste in Serbia-Attitudes, quantities and global warming potential. J. Clean. Prod..

[26] Comission for environmental Cooperation (2020), Retrieved from: http://www.cec.org/flwm/method/diaries/#:~:text=In%20a%20diary%20study%2C%20participants,might%20be%20given%20a%20scale.

[27] Block LG, Keller PA, Vallen B, Williamson S, Birau MM, Grinstein A, Haws KL, LaBarge MC, Lamberton C, Moore ES, Moscato EM (2016). The squander sequence: Understanding food waste at each stage of the consumer decision-making process. J. Public Policy Mark..

[28] EU, Refresh project (2016) Retrieved from: https://eu-refresh.org/

[29] Petty RE, Cacioppo JT (1986). The elaboration likelihood model of persuasion.

[30] Briñol P, Petty RE (2015). Elaboration and validation processes: Implications for media attitude change. Media Psychol..

[31] Levy N (2014). Consciousness, implicit attitudes and moral responsibility. Noûs.

[32] Flanagan A, Priyadarshini A (2021). A study of consumer behavior towards food-waste in Ireland: Attitudes, quantities and global warming potentials. J. Environ. Manag..

[33] Ajzen I (1991). The theory of planned behavior. Organ. Behav. Hum. Decis. Process..

[34] Graham-Rowe E, Jessop DC, Sparks P (2014). Identifying motivations and barriers to minimizing household food waste. Resour. Conserv. Recycl..

[35] Romani S, Grappi S, Bagozzi RP, Barone AM (2018). Domestic food practices: A study of food management behaviours and the role of food preparation planning in reducing waste. Appetite.

[36] Stancu V, Haugaard P, Lähteenmäki L (2016). Determinants of consumer food waste behavior: Two routes to food waste. Appetite.

[37] Graham-Rowe E, Jessop DC, Sparks P (2015). Predicting household food waste reduction using an extended theory of planned behaviour. Resour. Conserv. Recycl..

[38] Steinmetz J, Xu Q, Fishbach A, Zhang Y (2016). Being observed magnifies action. J. Personal. Soc. Psychol..

[39] Russell SV, Young CW, Unsworth KL, Robinson C (2017). Bringing habits and emotions into food waste behavior. Resour. Conserv. Recycl..

[40] DeCaro MS, Thomas RD, Albert NB, Beilock SL (2011). Choking under pressure: multiple routes to skill failure. J. Exp. Psychol. Gen..

[41] Faul F, Erdfelder E, Lang A-G, Buchner A (2007). G*Power 3: A flexible statistical power analysis program for the social, behavioral, and biomedical sciences. Behav. Res. Methods.

[42] Lally P, Van Jaarsveld CH, Potts HW, Wardle J (2010). How are habits formed: Modelling habit formation in the real world. Eur. J. Soc. Psychol..

[43] Lyndhurst. Retrieved from: https://www.wrap.org.uk/content/food-behaviour-consumer-research-quantitative-phase (2007).

[44] ForMat project. Downloaded from: https://ec.europa.eu/food/sites/food/files/safety/docs/fw_lib_format-rapport-2016-eng.pdf (2016).

[45] NRDC (2017). Estimating quantities and types of food waste at the city level Retrieved from: https://www.nrdc.org/sites/default/files/food-waste-city-level-report.pd

[46] Elimelech E, Ert E, Ayalon O (2019). Exploring the drivers behind self-reported and measured food wastage. Sustainability.

[47] van der Werf P, Seabrook JA, Gilliland JA (2020). Food for thought: Comparing self-reported versus curbside measurements of household food wasting behavior and the predictive capacity of behavioral determinants. Waste Manag..

