# Supplementary material for: Cerebrating and engagement, paths to reduce fresh produce waste within homes
Source: Sci Rep. 2024 Apr 6;14:8120. doi: 10.1038/s41598-024-58250-0 (PMC10998904; doi:10.1038/s41598-024-58250-0)
Supplement: Supplementary file 1 [file 41598_2024_58250_MOESM1_ESM.docx]

Table 1a: Mean and Standard deviations of fruit waste, in grams 

| Week | Mean | SD |
| --- | --- | --- |
| 1 | 172.9 | 273.7 |
| 2 | 177.6 | 332.4 |
| 3 | 141.7 | 272.5 |
| 4 | 156.1 | 279.0 |
| 5 | 114.9 | 211.4 |
| 6 | 118.0 | 221.1 |

Table 1b: Mean and Standard deviations of vegetable waste, in grams 

| Week | Mean | SD |
| --- | --- | --- |
| 1 | 239.5 | 352.7 |
| 2 | 261.0 | 394.3 |
| 3 | 237.4 | 434.3 |
| 4 | 183.8 | 300.3 |
| 5 | 220.8 | 406.8 |
| 6 | 186.3 | 271.3 |

Table 1a and b shows the means for both types of produce. It can be noted that both had a reduction in waste when looking over the 6-week period. However, only for fruit was the reduction significant. 

Table 2: Group comparisons across the main and follow on study for attitude measures

|  | Stance against | Neutral | Care about |
| --- | --- | --- | --- |
| Attitude | Follow on study | Main study | Follow on study | Main study | Follow on study | Main study |
| Environment | 42 | 25 | 47 | 55 | 11 | 20 |
| Ethics | 89 | 30 | 9 | 49 | 2 | 21 |
| Purchasing behaviours | 36 | 40 | 53 | 34 | 11 | 26 |
| Preparation | 9 | 13 | 37 | 26 | 54 | 61 |
| Not influenced by expiration date | 16 | 1 | 29 | 38 | 55 | 61 |

From the table it can be noted participants attitudes in the follow-on study was varied, this was also the case at the onset of the main study. Specifically, there were a large proportion of people that cared about preparation (54%) and were not influenced by expiration dates (55%). As these number were also high in the main study, it supports the idea that our original sample groups, even thought they had a large representation of participants that cared, is representative of the wider population. Mostly, the attitudes measured in this additional study is like those in the main study, except for ethics. 

Figure 1: Visual mapping of where in the UK participants lived

Map

Description automatically generated

Table 3: Demographic data for the participants

| Condition | Diary control (n = 40) | Diary intervention (n = 40) | Log control (n = 39) | Log intervention (n = 35) |
| --- | --- | --- | --- | --- |
| Age Mean (SD) | 44.3 (16.1) range 22-81 | 42.4 (12.1) range 21-72 | 41.9 (13.8) Range 19-77 | 39.3 (11.3) Range 19-60 |
| Gender | Male | 7 | 2 | 8 | 3 |
| Female | 33 | 38 | 31 | 32 |
| Relationship status | Single | 10 | 8 | 9 | 8 |
| Married no kids | 4 | 9 | 8 | 5 |
| Married with kids | 12 | 12 | 16 | 16 |
| In relationship no kids | 7 | 4 | 3 | 3 |
| In relationship with kids | 6 | 3 | 3 | 3 |
| Other | 1 | 4 | 0 | 0 |
| Income | Unemployed | 2 | 7 | 5 | 4 |
| < £15,000 | 6 | 8 | 16 | 6 |
| £15,000 - £34,999 | 22 | 7 | 10 | 13 |
| £35,000 - £54,999 | 4 | 10 | 3 | 9 |
| £55,000 – £74,999 | 6 | 5 | 1 | 2 |
| > £75,000 | 0 | 3 | 4 | 1 |
| No. of people in household | Just me | 8 | 6 | 6 | 6 |
| 2 | 12 | 17 | 13 | 12 |
| 3 | 10 | 9 | 5 | 7 |
| 4 | 9 | 6 | 12 | 8 |
| 5 or more | 1 | 2 | 3 | 2 |
| Education | Secondary | 8 | 5 | 7 | 7 |
| University degree | 17 | 21 | 22 | 15 |
| Post-graduate | 15 | 14 | 10 | 13 |
| Area of residence | Countryside | 2 | 0 | 2 | 2 |
| Village | 10 | 14 | 12 | 5 |
| Town | 15 | 12 | 14 | 14 |
| City | 13 | 13 | 11 | 14 |
| Does most of the cooking | Yes | 39 | 36 | 34 | 32 |
| No | 1 | 4 | 5 | 3 |
| Does most of the shopping | Yes | 37 | 37 | 38 | 34 |
| No | 3 | 3 | 1 | 1 |

n = Number of participants, SD = Standard deviation

Figure 2: Purchase log 

A picture containing table

Description automatically generated

Figure 3: Food diary

Table

Description automatically generated with medium confidence

Figure 4: Food waste log

Table, timeline

Description automatically generated
